# Comparative Transcriptome Analysis of Halophyte *Zoysia macrostachya* in Response to Salinity Stress

**DOI:** 10.3390/plants9040458

**Published:** 2020-04-04

**Authors:** Rong Wang, Xi Wang, Kuan Liu, Xue-Jie Zhang, Luo-Yan Zhang, Shou-Jin Fan

**Affiliations:** Key Lab of Plant Stress Research, College of Life Sciences, Shandong Normal University, Jinan 250014, China; 2017010077@stu.sdnu.edu.cn (R.W.); wangxi920108@163.com (X.W.); 2018020778@stu.sdnu.edu.cn (K.L.); zxjpublic@sohu.com (X.-J.Z.)

**Keywords:** *Zoysia macrostachya*, transcriptome response, salt stress, salt secretion

## Abstract

As one of the most severe environmental stresses, salt stress can cause a series of changes in plants. In salt tolerant plant *Zoysia macrostachya*, germination, physiology, and genetic variation under salinity have been studied previously, and the morphology and distribution of salt glands have been clarified. However, no study has investigated the transcriptome of such species under salt stress. In the present study, we compared transcriptome of *Z. macrostachya* under normal conditions and salt stress (300 mmol/L NaCl, 24 h) aimed to identify transcriptome responses and molecular mechanisms under salt stress in *Z. macrostachya*. A total of 8703 differently expressed genes (DEGs) were identified, including 4903 up-regulated and 3800 down-regulated ones. Moreover, a series of molecular processes were identified by Gene Ontology (GO) analysis, and these processes were suggested to be closely related to salt tolerance in *Z. macrostachya*. The identified DEGs concentrated on regulating plant growth via plant hormone signal transduction, maintaining ion homeostasis via salt secretion and osmoregulatory substance accumulation and preventing oxidative damage via increasing the activity of ROS (reactive oxygen species) scavenging system. These changes may be the most important responses of *Z. macrostachya* under salt stress. Some key genes related to salt stress were identified meanwhile. Collectively, our findings provided valuable insights into the molecular mechanisms and genetic underpinnings of salt tolerance in *Z. macrostachya*.

## 1. Introduction

As one of the most severe environmental stresses, salinity stress can cause a series of changes in plants. It has been reported that more than 20% of irrigated areas have been affected by salinity [1], and the situation is worsening [2]. Traditional irrigation mode can also cause salt accumulation in soil, leading to negative effects on modern civilization [3]. Nowadays, many types of crops are sensitive to salt stress. Therefore, it is urgently necessary to develop an economic and effective way to enhance the salt tolerance of plants.

Salinity can impact the growth and development of plants. Salt stress has an effect on production of reactive oxygen species (ROS). To avoid damages resulting from ROS, higher plants have developed a complex ROS scavenging system [4,5]. ROS scavenging system efficiency is crucial for cell resistance to ROS-mediated injury [6,7,8]. The activities of antioxidant enzymes were increased by NaCl in many plants [4,9,10,11,12]. It is reported that the enhancement of antioxidant enzymes activity can improve salt tolerance in plants [13,14,15,16]. In addition, salinity can cause hyperosmotic stress and ion disequilibrium [17]. Plants can reduce these effects by reducing accumulation of toxic ions in the leaf blades (Na^+^ and Cl^−^ exclusion) [18]. Salt glands have been reported to be important in salt secretion under salt stress [19,20,21]. Several ion transporters have been discovered to play critical roles in the process of salt secretion [22,23]. Yuan et al. have found that some candidate genes associated with ion transport, vesicular transport, and ROS scavenging have a close relationship with salt gland secretion [24]. Besides, plant hormones are also strongly associated with abiotic stresses. Plant hormones play fundamental roles when plants are exposed to many different abiotic stresses, including drought, heat, cold, and salinity [25,26,27]. Among them, major hormones are auxins, gibberellins (GAs), cytokinins (CKs), and abscisic acid (ABA). These hormones not only regulate plant growth, but also protect plants from abiotic stresses, of which ABA is an important element in signal transduction under stressful conditions.

*Zoysia macrostachya* Franch. and Sav. is a perennial plant, belonging to Chloridoideae, Poaceae [28,29,30]. It is a kind of wild beach plant and widely distributed in Japan, Korea and the eastern coastal area of China [31,32,33,34,35]. It always loosely spreads from extensive, deep, slender rhizomes. This species can be used as a forage plant and lawn grass because of its high reproductive capacity and rich nutrition value [36,37]. No commercial cultivars have been developed. It is a type of C_4_ grass and can have a normal growth even under serious stresses [38,39]. This species has a wide adaption capacity to different environments and possesses a high tolerance to salt. Previous studies have paid attention to germination, physiology, genetic variation, and landscaping. Zhao et al. [40] found salinity conditions can have an influence on seed’s germination of *Z. macrostachya* and measured the critical and limit concentration of salt tolerance in this species. Zhao et al. [41] showed plant average height, fresh weight, and dry weight declined with the increase of NaCl concentration. Hu et al. [42] and Zhang et al. [43] found the content of soluble sugar, proline, and the activity of POD (peroxidase) are increasing in the portion of curled leaves in *Z. macrostachya* under NaCl condition. Li et al. [44] and Zhao et al. [45] compared responses of three *Zoysia* grass species to salt stress and discovered an increase of Na^+^ content, relative conductance of leaf tissues, and MDA (malondialdehyde) content under salt stress. Relative conductance of tissues can be used to estimate membrane damage. The MDA content was found to increase slower growth in *Zoysia macrostachya* than the other two species. So, they considered this species has greater salt resistance than the other two species. As a typical exo-recretohalophyte, it has salt glands on its epidermis [46]. Different responses of *Z. macrostachya* at the gene expression level have not been reported under salt stress. *Z. macrostachya* can be used as a candidate plant to deal with salt stress, screen salt-related genes, and study molecular mechanisms of halophytes. 

Transcriptional profiling analyses have been used as important methods to reveal changes of plants at the gene expression level under salt stress [47,48,49,50]. Salt resistance is not determined by a single gene, which requires many genes to work together. Many species have been analyzed, such as Arabidopsis (Columbia) [51], barley and rice [52], and *Limonium bicolor* [24]. Many biological processes were found to be related to salt stress through transcriptome analysis, such as photosynthesis [53,54], glycolysis/gluconeogenesis [55,56], and ROS scavenging system [57,58]. Wang et al. found that the auxin signal transduction family, ABA (abscisic acid) signal transduction family, WRKY TF family, and bHLH TF family may be the most important families in *Zoysia* salt-stress regulation, but the molecular regulatory mechanism remains unknown [59]. 

In the present study, we aimed to reveal the salt-induced responses of *Z. macrostachya* via transcriptome sequencing of untreated and NaCl-treated (300 mmol/L, 24 h) plants. Many differently expressed genes (DEGs) were identified in *Z. macrostachya* under salt stress, and some biological processes significantly correlated with salt stress were determined by Gene Ontology (GO) analysis. In addition, we also investigated the salt-tolerance and adaptation mechanisms of *Z. macrostachya*.

## 2. Results

### 2.1. Plant Materials and Growth Condition

**Figure 1:**
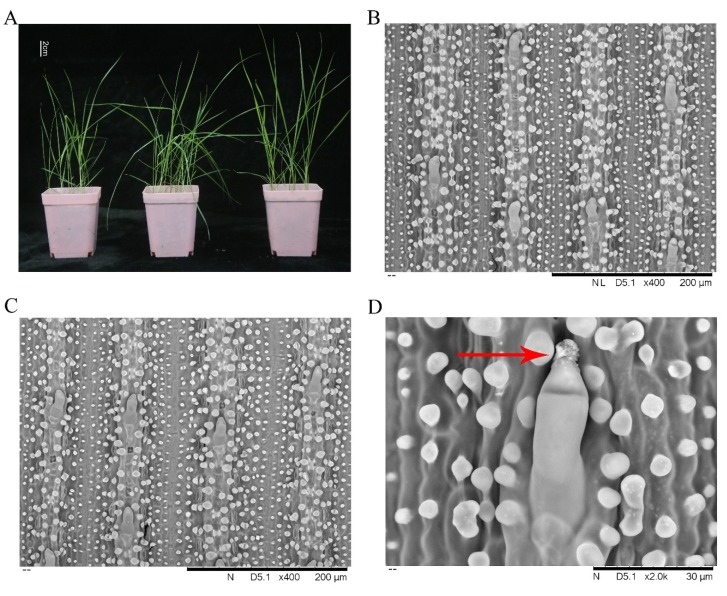
Salt secretion and ultrastructure characteristics of salt glands (microhairs) in *Z. macrostachya* under SEM. A salt gland of *Z. macrostachya* consists of a pair of cells. The longer cell located in the lower position is basal cell, the approximately triangular cell at the top is cap cell. (**A**) seedlings of *Z. macrostachya* (2-month-old). (**B**) distribution of salt glands on adaxial surfaces in control group. (**C**) distribution of salt glands on adaxial surfaces in NaCl-treated group. (**D**) salt glands on adaxial surfaces under 300 mmol/L NaCl. The arrow points to a grain of crystal.

Figure 1A shows the 2-month-old seedlings of *Z. macrostachya* before treatment. Plants grow vigorously and have green leaves. Culms were ascending, branched at ground level. Leaf sheaths were glabrous. Leaf blades were linear-lanceolate, narrow, and flat. We did not observe some significant changes after 300 mmol/L NaCl for 24 h visually. 

### 2.2. SEM (Scanning Electron Microscope) Observation and Energy Spectrum

Figure 1D shows that a grain of crystal was observed from the salt gland of *Z. macrostachya*. A salt gland consists of a pair of cells. The longer cell located in the lower position is the basal cell, the approximately triangular cell at the top is the cap cell. Salt glands are distributed on both sides of the leaf blade. The element (Na, K, Ca, Cl) content (weight %) of salt gland cells was determined by energy spectrum. Table 1 lists the results.

### 2.3. Measurement of Biochemical Parameters

**Figure 2:**
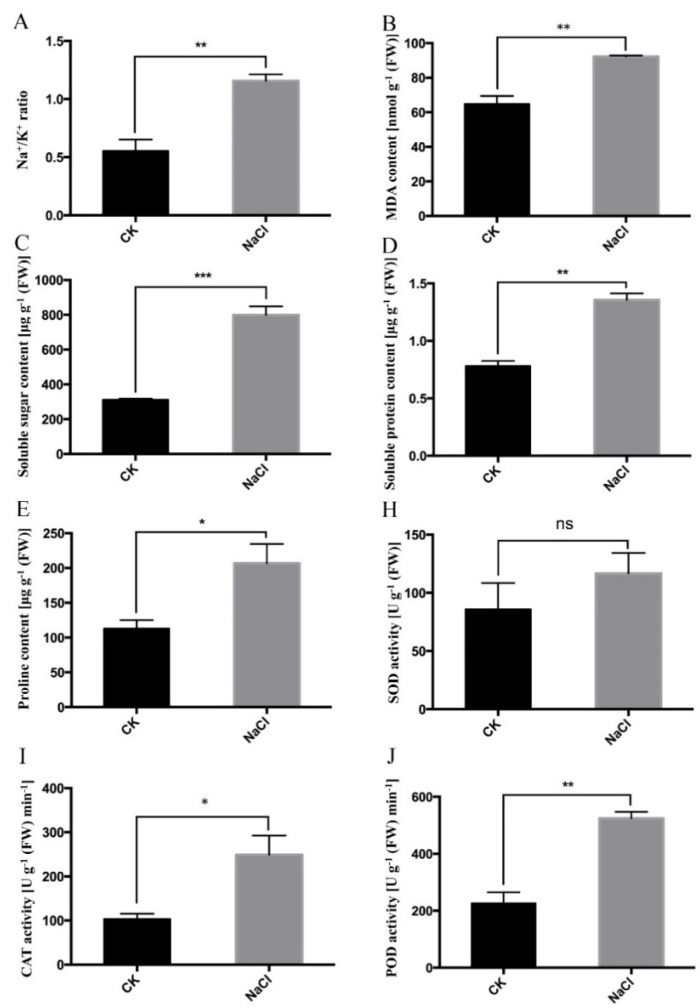
Biochemical parameters of leaf tissue in control group and 300 mmol/L NaCl-treated group (24 h), including Na^+^/K^+^ ratio, malondialdehyde (MDA) content, soluble sugar content, soluble protein content, proline content, superoxide dismutase (SOD) activity, catalase (CAT) activity, and peroxidase (POD) activity. The data were obtained by averaging three biological replicates. The error bars represent ± SE. *n* = 3. *: *p* < 0.05, **: *p* < 0.01, ***: *p* < 0.001, ns: not significant.

As shown in Figure 2, we examined eight biochemical parameters of control group and treatment group, including SOD (superoxide dismutase), POD, CAT (catalase), MDA, soluble sugar, soluble protein, proline, and Na^+^/K^+^ ratio, and found that there was a significant increase in terms of these parameters in the 300 mmol/L NaCl-treated group compared with the control group.

### 2.4. Transcriptome Sequencing

**Figure 3:**
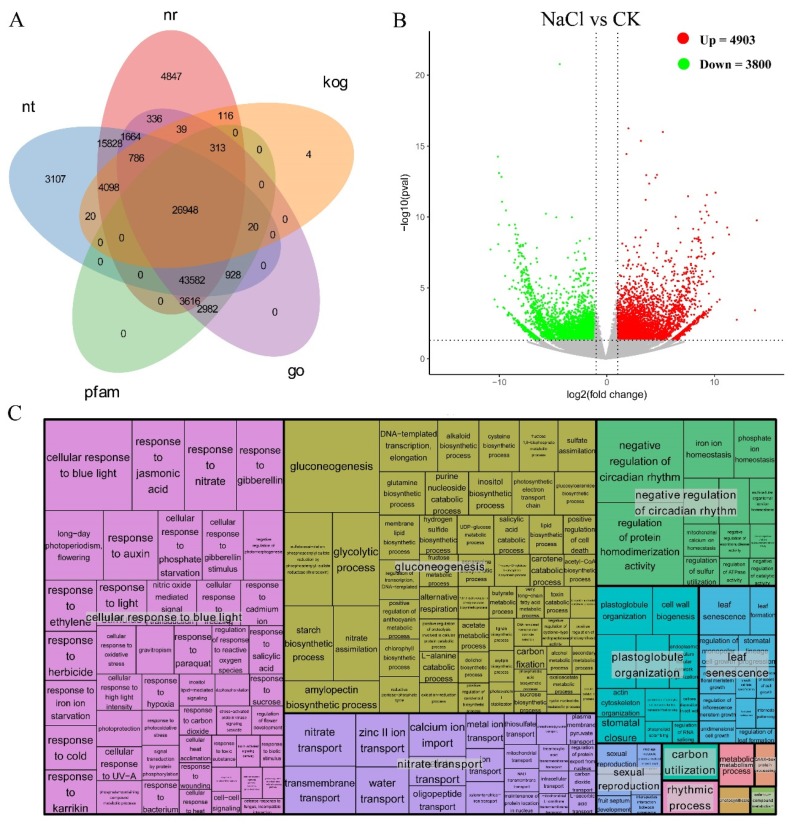
(**A**) Venn diagram of functional annotations of unigenes in Nt (NCBI non-redundant protein sequences), Nr (NCBI non-redundant protein sequences), KOG (Clusters of Orthologous Groups of proteins), GO (Gene Ontology) and Pfam (Proteinfamily) databases. (**B**) Volcano figure of up-regulated and down-regulated unigenes in *Z. macrostachya*. (**C**) REVIGO analysis of up-regulated differently expressed genes (DEGs) in *Z. macrostachya*. Each rectangle is a single cluster representative. The representatives are joined into “superclusters” of loosely related terms, visualized with different colors. Size of the rectangles was adjusted to reflect the *p* value of the GO term calculated by TopGO.

After sequencing and filtering, 56,447,208, 46,646,078, and 54,159,280 clean reads were respectively obtained from three samples in the control group, and 51,334,336, 61,039,822, and 56,731,060 clean reads were respectively obtained from three samples in the treatment group. Under the optimal assembly conditions, assembly generated 130,636 unigenes with a N50 (the scaffold length such that 50% of the assembled sequences) of 2602. Among them, more than 92.45% unigenes had a quality score of 30 (Q30) and an error probability of 0.1%. Gene function was annotated based on seven databases, including Nr (NCBI non-redundant protein sequences), Nt (NCBI non-redundant nucleotide sequences), Pfam (protein family), KOG/COG (clusters of orthologous groups of proteins), Swiss-Prot (a manually annotated and reviewed protein sequence database), KO (KEGG ortholog database), and GO (gene ontology). A total of 130,636 unigenes were successfully annotated in these databases. Figure 3A illustrates the result of annotated unigenes in five databases.

### 2.5. Differently Expressed Genes (DEG) Analysis

A total of 8703 DEGs were identified according to differential gene expression analysis, including 4903 up-regulated DEGs and 3800 down-regulated ones in the NaCl-treated group (*p* value < 0.05 and |log_2_ (fold change)|>1) (Figure 3B). The most significantly up-regulated unigene was Ntn-hydrolase superfamily protein (Cluster-8095.49787, L_2_fc = 7.2493), while the most down-regulated one was RNA polymerase sigma-subunit C (Cluster-8095.72925, L_2_fc = −4.2799). Among these DEGs, 768 were specifically expressed in the NaCl-treated group, and 750 were specifically expressed in the control group. Information of the up- and down-regulated unigenes in *Z. macrostachya* can be seen in Table S2.

### 2.6. Go (Gene Ontology) Enrichment Analysis

To confirm molecular mechanisms of *Z. macrostachya* under salinity conditions, the DEGs were subjected to GO classification. A total of 276 Go terms, such as “gluconeogenesis” (GO: 0006094), “cellular response to blue light” (GO: 0071483), and “negative regulation of circadian rhythm” (GO: 0042754), were enriched among up-regulated DEGs. Meanwhile, 210 Go terms, such as “RNA modification” (GO: 0009451), “RNA secondary structure unwinding” (GO: 0010501), and “circadian rhythm” (GO: 0007623), were enriched among down-regulated DEGs. 

### 2.7. qRT-PCR (Quantitative Real-Time PCR)

**Figure 4:**
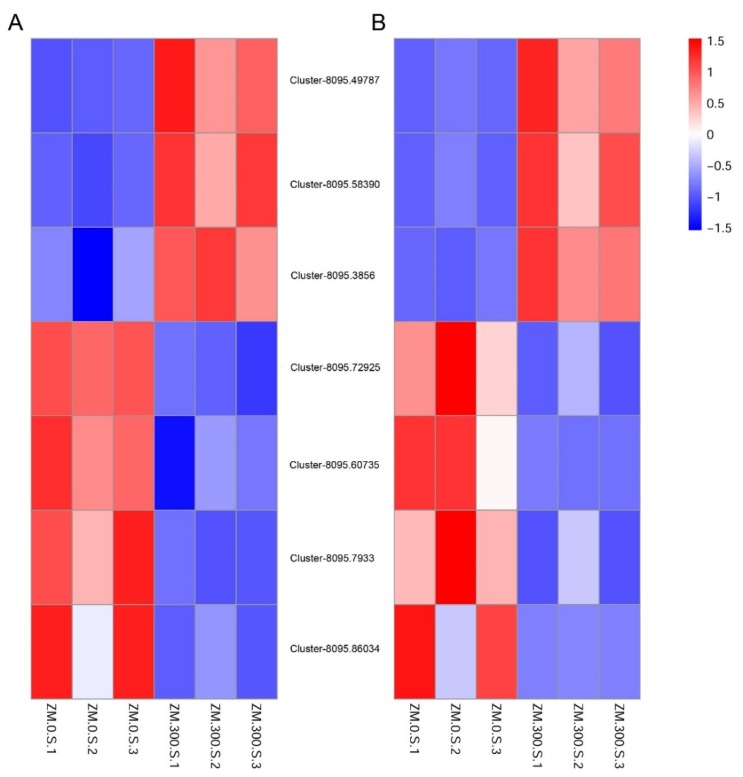
qRT-PCR verification of 7 DEGs of *Z. macrostachya* under NaCl treatment. (**A**) represents RNA-seq data. (**B**) represents qRT-PCR data. Different rows represent different unigenes, and different colors represent values of log2 to indicate different gene expression level. ZM.0.S.1, ZM.0.S.2, and ZM.0.S.3 represent shoots of *Z. macrostachya* under 0 mmol/L NaCl, ZM.300.S.1, ZM.300.S.2, and ZM.300.S.3 represent shoots of *Z. macrostachya* under 300 mmol/L NaCl. A red color indicates that the gene is highly expressed under the corresponding treatment. A blue color indicates that the gene is lowly expressed under the corresponding treatment. Color bar indicates means Log2() value of FPKM for unigenes.

qRT-PCR was carried out to assess the expressions of DEGs. Three up-regulated unigenes (Cluster-8095.49787, Cluster-8095.58390, and Cluster-8095.38560) and four down-regulated unigenes (Cluster-8095.72925, Cluster-8095.60735, Cluster-8095.7933, and Cluster-8095.86034) were selected to verify the RNA-seq (a high-throughput cDNA sequencing method) results. The result showed that these genes had a similar expression pattern between these two methods (Figure 4). 

## 3. Discussion

Plant growth is seriously affected by soil salinization, and only a few plants can survive under high-salinity stress. It is a complex process for plants to respond to abiotic stress. Several genes have been reported to be associated with salt tolerance. However, the molecular mechanism underlying salt tolerance in plants remains largely unexplored. The seashore halophyte *Z. macrostachya* can adapt to high salinity and even survive in sea water. Previous studies of *Zoysia* species have demonstrated that they can expel excess salt to extracellular environment via salt glands. In our current study, we analyzed RNA-seq data of control group and 300 mmol/L NaCl treatment group. A total of 4903 up-regulated and 3800 down-regulated unigenes were identified in *Z. macrostachya*. Some transcriptome changes were found in plants under saline conditions in *Z. macrostachya*, revealing some potential mechanisms for salt tolerance.

### 3.1. Plant Growth and Hormone Signal Transduction

Plant hormones can regulate the growth and development of plants, playing key roles in salt stress response and adaptation [60]. Moreover, many proteins have been shown to be involved in signaling transduction pathways of plant hormones, such as receptors and transcription factors [61,62,63,64].

A great deal of evidence indicated that auxin was involved in response to salinity stress in plants, while little was known about its underlying mechanism [65,66,67]. Auxin accumulation and redistribution lead to a series of changes in plants under salinity stress. A previous study has suggested that the plastic development of root system architecture is modulated by auxin redistribution under salt stress in *Arabidopsis thaliana* [65]. It is a fundamental process to import auxin to control a multitude of plant development. It has been demonstrated that AUX1 is a high-affinity IAA importer, and we found that its associated unigenes were up-regulated. Yang et al. [68] have shown the similar results. The AUX/IAA genes encode the Aux/IAA proteins and are regulated by auxin. AUX/IAA is a big gene family and shares four conserved amino-acid sequence motifs, which are called domains I, II, III, and IV. Many studies have indicated that Aux/IAA proteins modulate the gene expression by interacting with auxin response factors (ARF) proteins to change their activities [69]. Aux/IAA protein and auxin response factors (ARFs) are important regulators in auxin signaling. These short-lived nuclear proteins can help plants sense and respond to changes in auxin levels quickly [70]. Overexpression of Aux/IAA genes can improve salt tolerance, such as grapevine *VvIAA18* gene [71]. We also observed cessation of plant growth when they were treated with NaCl, and such findings were consistent with auxin-mediated regulation in plants. In our present study, we found unigenes related to auxin transporter-like protein 1 (LAX1, Cluster-8095.51066, L_2_fc = 1.3724) and AUX/IAA transcriptional regulator family protein (Cluster-19750.1, L_2_fc = 2.5188; Cluster-8095.68690, L_2_fc = 1.4516). These genes were up-regulated under salt stress. 

GAs generally participate in seed germination, leaf expansion, photomorphogenesis, stem elongation, flowering, and so on [72,73]. However, rice has only one DELLA protein (Slender Rice1 SLR1), while the Arabidopsis genome encodes five DELLAs (GA-insensitive GAI, repressor of GA1-3 RGA, RGA-like1 RGL1, RGL2, and RGL3), all of which share the DELLA-motif in their N-terminal domain, as well as the C-terminal GRAS conserved domain [74,75]. DELLA family proteins are major GA negative regulators [76]. It has been reported that salt stress causes DELLA accumulation through reducing the GA content by elevating the GA-2-oxidase activity [77]. In our present study, unigenes associated with DELLA proteins were up-regulated. We inferred that accumulation of DELLA protein was caused by a rapid reduction of GAs, leading to enhanced salinity tolerance. Moreover, “response to gibberellin” (GO: 0009739), “cellular response to gibberellin stimulus” (GO:0071370), and “Gibberellic acid mediated signaling pathway” (GO: 0009740) were significantly enriched by up-regulated genes, such as GRAS family transcription factor family protein coding gene *GAI* (Cluster-8095.57900, L_2_fc = 1.0677), GA-regulated family protein (Cluster-8095.19881, L_2_fc = 1.7948; Cluster-8095.19882, L_2_fc = 2.4827), and GA-regulated protein GASA4 (Cluster-8095.75935, L_2_fc = 6.8393).

As a plant hormone, ABA plays a critical role in mediating the seed germination and development of plants [78,79,80]. Meanwhile, ABA is a fundamental element in plant signal transduction under stress [81,82,83,84]. It can modulate several physiological processes, including seed dormancy, development, and responses to biotic and abiotic stresses by activating a complex signaling network [85]. Abiotic stresses always result in enhanced biosynthesis and accumulation of ABA. The content of ABA is increased under stressful conditions, maintaining the water status of plants [66]. Enzymes associated with ABA biosynthesis have been determined in *Arabidopsis thaliana* [86]. ABA-binding proteins are located on cell membrane, cytosol, and many different positions in the cell. Therefore, many researchers believe that there are many different types of ABA receptors. So far, many ABA receptors have been recognized, while little detailed information is known. Among them, pyrabactin resistance (PYR)-like (PYL)/regulatory component of ABA receptor (RCAR) family ABA receptors always connect with many ABA regulators to regulate ABA signal transduction. PYR/PYLs belong to the star-related lipid-transfer (START) protein family, and they can recognize ABA signal and activate downstream signaling. When endogenous ABA exists, PYR/PYLs interact with PP2Cs, suppress phosphatase activity, activate SnRK2 and phosphorylate target proteins [87]. Under stressful conditions, the increased ratio of PP2Cs:PYR/PYLs may be necessary for activation of the downstream ABA signaling pathway, such as RCAR3 (Cluster-8095.38285, L_2_fc = 8.2229), protein phosphatase 2C family protein (Cluster-8095.5592, L_2_fc = 6.3361; Cluster-8095.80867, L_2_fc = 7.5602; Cluster-8095.81571, L_2_fc = 1.0973), and PYR1-like 4 (Cluster-8095.81571, L_2_fc = 1.7234). 

### 3.2. Maintaining Ion Homeostasis via Salt Secretion and Accumulation of Osmoregulatory Substances

A large amount of water enters plant cells, therefore many halophytes show succulent leaves in salinity environment [88,89,90]. However, such a situation was not observed in *Z. macrostachya*, indicating that there was a different mechanism for *Z. macrostachya* to respond to high salinity. Previous studies have observed bicellular salt glands in *Zoysia* species, lying parallel to the intercostal ridge on the leaf surface [91]. Microhairs have been detected in many grass species, except for Pooideae [92,93], and they only function as salt glands in Chloridoideae [19,22,94,95]. Several unigenes associated with trichome differentiation were determined as DEGs, which might be related to the formation and development of salt glands, such as a coiled-coil domain protein (Cluster-5467.0, L_2_fc = 2.6091; Cluster-5467.2, L_2_fc = 3.5991), a dynamin-like protein (Cluster-8095.35582, L_2_fc = 7.3026), and fdiester phosphodiesterase-like protein (Cluster-8095.53790, L_2_fc = 2.6538; Cluster-8095.62103, L_2_fc = 1.0037; Cluster-8095.77020, L_2_fc = 1.5168). Therefore, a hypothesis has been raised that salinity tolerance of *Zoysia* species may be associated with ion exclusion via leaf salt glands [96]. In the present study, we did not observe visually essential differences between the control group and NaCl treatment group in salt gland density. However, glands are better developed on the adaxial surfaces [91]. Salt glands in *Zoysia* are always called microhairs, which are mainly distributed on two sides of leaves. Figure 1 shows that there is a grain of crystal on the top of the salt gland cap cell of *Z. macrostachya*. Energy spectrum showed that Na and Ca were not detected in basal cells of the control group, and these two elements (Na and Ca) were significantly increased in the NaCl treatment group compared with the control group. In the NaCl treatment group, the content of Na in basal cells was much higher compared with the cap cells. In our present study, ion transporter related genes were up-regulated under NaCl treatment, such as membrane-localized and endosomal Na^+^/H^+^ antiporters (Cluster-8095.61639, L_2_fc = 4.3726; Cluster-8095.90290, L_2_fc = 6.3834), potassium channel protein KAT1 (Cluster-8095.22112, L_2_fc = 7.4757), and chloride channel protein (Cluster-8095.13407, L_2_fc = 2.2973; Cluster-8095.21459, L_2_fc = 1.0952). Na^+^/H^+^ antiporter SOS1 was identified to mediate the efflux of Na^+^ from root cells into the soil or cortical apoplast [97,98]. Du et al. found *ZjNHX1* (Na^+^/H^+^ antiporter gene) was up-regulated in *Zoysia japonica* after NaCl treatment [99]. Na^+^/H^+^ antiporters may play an important role in salt tolerance and ion homeostasis in *Zoysia* species. It is reported that overexpression of Na^+^/H^+^ antiporters related genes can improve salt tolerance in *Arabidopsis thaliana* [100]. 

Figure 2 shows that Na^+^/H^+^ ratio increased in 300 mmol/L NaCl-treated leaf tissues. The capacity of plants to maintain a low cytosolic Na^+^/K^+^ ratio is likely to be one of the key determinants of plant salt tolerance [101,102]. The balance of these two ions in plant cells will depend on the concerted action of transport systems located at plasma and vacuolar membranes. Besides, the accumulation of proline, soluble sugar and soluble protein is an important way to enhance osmotic potential of plant cells to keep ion equilibrium [103]. In our study, the contents of proline, soluble sugar, and soluble protein increased in the NaCl treatment group compared with the control group [100]. 

### 3.3. Oxidative Stress Regulation and ROS (Reactive Oxygen Species) Scavenging System 

Saline stress can result in a series of physiological alternations in plants. The production of ROS reflects an early response to salt stress [25], and the activities of antioxidant enzymes have been shown to elevate under saline conditions [104,105,106]. The increase of MDA content indicates membrane lipid peroxidation and a membrane damage in cells [107,108]. Plants can protect themselves from harmful oxidative reactions through their efficient ROS scavenging systems, including enzymatic and non-enzymatic antioxidative systems, such as SOD, CAT, and POD. GO terms of “cellular response to oxidative stress” (GO: 0034599), “response to reactive oxygen species” (GO: 0000302), and “regulation of response to reactive oxygen species” (GO: 1901031) were enriched by up-regulated genes. The expressions of some ROS responsive proteins were found to be significantly increased, such as a chloroplastic thylakoid ascorbate peroxidase tAPX (Cluster-8095.43497, L_2_fc = 1.941), cytosolic ascorbate peroxidase APX1 (Cluster-8095.44903, L_2_fc = 3.9559), and plasma membrane-localized Na^+^/H^+^ antiporter SOS1 (Cluster-8095.61639, L_2_fc = 4.3726). The activities of these enzymes were enhanced under NaCl treatment, indicating that stress caused ROS accumulation in cells and might damage cellular components. Therefore, the plant enhanced enzyme activity to scavenge ROS. The increased enzyme activity has been reported in many plants, such as salt-tolerant genetype cotton [109], alfalfa [110], *Suaeda salsa* [111], *Cakile maritime* [112], and mangrove [113], while there is a decrease or no difference in some salt-sensitive plants [114,115].

Overexpression of ROS scavenging system related genes can improve tolerance of plants to abiotic stresses. Sun et al. [116] introduced the thylakoid-bound ascorbate peroxidase gene from tomato leaf (*TtAPX*) into tobacco and improved the photochemical efficiency of photosystem 2 in tobacco. The reactive oxygen scavenging system can act as an intermediate link between correlative factors and plant stress resistance. Ethylene, cytokinin, and even fungi can modulate ROS homeostasis to regulate plant resistance [117,118,119]. Saurabh et al. suggested that ectopic overexpression of *AtApx1* gene can confer salt tolerance by strengthening ROS scavenging system [120]. This system may be a starting point for improving plant salinity.

## 4. Materials and Methods 

### 4.1. Plant Material and Treatment

Mature seeds of *Z. macrostachya* were collected in Binzhou, Shandong, China (N38°15′38.46″, E117°52′11.43″, 2017.10.15). These seeds were stored in seed bags at 4 °C before germination. Seeds were immersed in water for 2 days, treated with 20% NaOH for 25 min, and then washed with distilled water. Clean seeds were germinated at 25 °C on moist filter paper in petri dishes. Seeds were placed in an electro-thermal incubator. Distilled water was regularly added to the filter paper. After germination, the seedlings were transferred to plastic pots (7 cm × 7 cm × 7.5 cm) filled with a perlite/peatmoss/vermiculite (1:1:1 *v*/*v*) mixture in greenhouse under the conditions of 15/9-h light/dark cycle at temperature (28 ± 3/20 ± 3 °C, day/night) with illumination of 600 μmol/m^2^s^−1^. Each pot of 6 plants and three pots each group, irrigated with 1/2 hoagland nutrient solution every day. 2-month-old seedlings were used in this experiment. Individuals with the same growth status were used for treatment. Control group was irrigated with distilled water, while NaCl-treated group was irrigated with 300 mmol/L NaCl solution. The irrigation solution was poured until the liquid was running out. Seedlings treated with the same concentration were placed in a large pot and excess liquid flows out into the pot. Leaf tissue was collected in 5 mL tube after 24 h salt treatment, immediately frozen in liquid nitrogen and stored at −80 °C prior to sequencing analysis. A moderate amount of leaf blade was stored in FAA solution for Scanning electron microscopy observation. Leaf tissue for biochemical parameters measurement were collected in 2 mL tube after weighting. 

### 4.2. Scanning Electron Microscopy (SEM) and Energy Spectrum 

To observe microhair changes of *Z. macrostachya* between the control group and NaCl-treated group, different positions (upper, middle, and lower) of leaf tissue were collected for SEM observation. Leaves of control group and NaCl-treated group were cut into small pieces and fixed in 2.5% glutaraldehyde solution. Samples were stored in 4 °C for 24 h, glutaraldehyde solution was replaced twice during the period of storage. Samples were washed with 0.1 mol/L phosphoric acid buffer for four times, fixed with 1% osmic acid for 1.5 h, then rinsed with deionized water and dehydrated with different concentrations of ethanol (30%, 50%, 70%, 85%, 95% for 15 min for each concentration). Then samples were treated with 100% ethanol twice, 20 min each time. Moreover, the samples were soaked in isoamyl acetate for 30 min and dried at the conventional critical point (E3100, Quorum, UK) [121,122]. Materials were positioned on the sample stand, and metal spraying (SC7620, Quorum, UK, 2 min) was carried out before observation. SEM was performed on Hitachi TM3030. Energy spectrum was used to examine element content (weight %) of leaf tissue. 

### 4.3. Measurement of Biochemical Parameters

The leaves without NaCl treatment were used as the control group. Treatment group was treated with 300 mmol/L NaCl for 24 h. There are three biological replicates in both the control group and the NaCl-treated group, each of which was measured three times. Levels of SOD, POD, soluble sugar, soluble protein, and proline were determined by nitroblue tetrazolium (NBT) method, guaiacol method, anthrone colorimetric method, Coomassie brilliant blue G-250 method, and sulfosalicylic acid method, respectively [123,124,125,126,127,128]. Activity of catalase (CAT) was determined using quartz cuvette by hydrogen peroxide assay [129]. MDA content measurement referred to other articles [130]. Flame spectrophotometer (M410, Sherwood, UK) was used to determine Na^+^ and K^+^ contents. 

Then, 0.15 g leaf tissues were rapidly ground into powder in liquid nitrogen. 1.5 mL of pre-cooled phosphate buffer with pH of 7.8 was added to rinse the mortar and transferred to the 2 mL EP tube. The supernatant was collected after centrifugation at 4000 r/min at 4 °C for 15 min. The supernatant was used to measure the activity of SOD, POD, CAT, MDA content, and soluble protein content. 3 mL of SOD reaction solution (0.5 mmol/L phosphate buffer: 130 mmol/L methionine: 750 μmol/L NBT: 100 μmol/L EDTA-Na_2_: 20 μmol/L riboflavin: H_2_O = 15:3:3:3:3:2.5, in order) and 20 μL of enzyme solution were mixed in test tubes and placed under 4000 Lux for 30 min. 20 μL of phosphate buffer was placed in a dark place as a blank sample, 20 μL of phosphate buffer mixed with 3 mL of SOD reaction solution was placed in a dark place as a control sample. Absorbances at 560 nm of the solution were used to calculate SOD activity (Ultraviolet spectrophotometer, T6, Puxi, China). 20 μL of enzyme solution and 3 mL POD reaction solution (0.1 mol/L phosphate buffer + 28 μL guaiacol + 19 μL 30% H_2_O_2_) were mixed, absorbances at 470 nm of the solution were used to calculate POD activity, values were read every 30 s for 5 times. 3 mL of reaction solution (0.1 mol/L phosphate buffer: 0.1 mol/L H_2_O_2_ = 1:4) and 0.1 mL of enzyme solution were mixed, phosphate buffer was used as control, the absorbance at 240 nm were read every 30 s for 5 times. Values were used to calculate CAT activity. 0.4 mL supernatant was mixed with 1.6 mL 0.5% thiobarbituric acid (TBA), and incubated at 95 °C for 30 min. Samples were centrifuged at 4000 r/min for 10 min at 4 °C and standing at room temperature for 15 min, absorbances at 532 and 600 nm of the solution were used to calculate MDA content. 0.1 mL of the enzyme solution and 0.9 mL of deionized water were mixed in test tubes. Deionized water was used as control. 5 mL of Coomassie brilliant blue G-250 was added to each tube, and the absorbance values at 595 nm were determined after fully mixing. Values were used to calculate soluble protein. 

Standard curves were required for soluble sugar and proline measurements. 0.2 g leaf tissues and 10 mL of deionized water were placed in test tubes and bathed in boiling water for 15 min. The upper liquid was extracted and transferred to 25 mL brown volumetric bottles. Deionized water was added into 25 mL. 0.5 mL of leaf extract solution, 1.5 mL of deionized water, 5 mL of concentrated sulfuric acid, and 0.5 mL of ethyl anthranone acetate solution were mixed and boiled in water for 1 min. The absorbance value at the wavelength of 620 nm was determined with blank as the control after cooling. Values were used to calculate soluble sugar content. 0.2 g leaf tissues and 5ml of 3% sulfosalicylic acid solution were placed in test tubes and bathed in boiling water for 10 min. After cooling, the filtrate was taken, 0.5 mL of extracting solution, 2 mL of ninhydrin, and 2 mL of acetic acid were mixed and bathed in boiling water for 30 min. After cooling, 4 mL of methylbenzene was added in test tubes. The upper solution was absorbed, toluene was used as the blank control, and the absorbance was measured at 520 nm. Values were used to calculate proline content.

### 4.4. Transcriptome Sequencing

Total RNA was isolated from three biological replicates of control group and NaCl-treated group using RNAprep Pure Plant Kit (Polysaccharides and Polyphenolics-rich, Tiangen, Beijing, China). Degradation, contamination, purity, concentration, and integrity of purified RNA were examined to ensure the quantification and qualification. RNA degradation and contamination were monitored on 1% agarose gels. RNA purity was checked using the NanoPhotometer^®^ spectrophotometer (Implen, Westlake Village, CA, USA). RNA concentration was measured using Qubit^®^ RNA Assay Kit in Qubit^®^ 2.0 Flurometer (Life Technologies, Carlsbad, CA, USA). RNA integrity was assessed using the RNA Nano 6000 Assay Kit of the Agilent Bioanalyzer 2100 system (Agilent Technologies, Santa Clara, CA, USA). Subsequently, NEBNext^®^ Ultra™ RNA Library Prep Kit for Illumina^®^ (New England Biolabs, Ipswich, MA, USA) was used to generate sequencing libraries. Illumina Hiseq platform was employed to sequence the libraries and paired-end reads were generated. All the transcriptome sequence data were submitted to the NCBI Sequence Read Archive (SRA) database (https://www.ncbi.nlm.nih.gov/sra), SRA accession: PRJNA600364.

### 4.5. Quality Control and Transcriptome Assembly

Adapter-containing and poly-N reads as well as low-quality reads were removed from raw data to obtain clean data, which were used in all the subsequent analyses. All the downstream analyses were based on clean data with high quality. De novo transcriptome assembly was conducted using Trinity (r20140413p1) [131] with min_kmer_cov set to 2 and other parameters of default settings. 

### 4.6. Identification of DEGs

DEGs between control group and NaCl-treated group were identified using DESeq R package (1.10.1). The RSEM (RNA-seq by expectation maximization) method was used to calculate gene expression level in our study [132]. *p*-value < 0.005 and |log_2_(fold change)|>1 were set as the threshold for significantly differential expression.

### 4.7. Go Enrichment Analysis

GO analysis was carried out to evaluate the functional and biological implications of DEGs using GOseq R packages [133].

### 4.8. qRT-PCR

Seven DEGs under salinity stress were examined by qRT-PCR. Among these DEGs, three were up-regulated in the treatment group, including Cluster-8095.49787, Cluster-8095.58390, and Cluster-8095.38560, and four were down-regulated, including Cluster-8095.72925, Cluster-8095.60735, Cluster-8095.7933, and Cluster-8095.86034. The shoots of the salt-treated plants were sampled to examine the relative expression of DEGs. Total RNA was isolated using Quick RNA Isolation Kit (waryong, Beijing, China), and then 500 ng purified RNA was reversely transcribed into cDNA using a QuantiTect Reverse Transcription Kit (Qiagen, Crawley, UK). The cDNA was diluted before qRT-PCR. Primers were specifically designed to span exon-exon junctions. PCR was performed in a 20-μL reaction system consisting of 2 μL template, 6.8 μL ddH_2_O, 0.6 μL of each primer, and 10 μL SYBR Green. Three biological replicates with three analytical replicates each were performed in qRT-PCR experiments.

## 5. Conclusions

In the present study, we compared transcriptome data of *Z. macrostachya* between the control group and 300 mmol/L NaCl treatment group. A total of 8703 DEGs were identified, including 4903 up-regulated and 3800 down-regulated ones. A series of molecular processes were identified by GO analysis, and such processes were suggested to be closely related to salt tolerance in *Z. macrostachya*. The identified DEGs concentrated on regulating plant growth via plant hormone signal transduction, maintaining ion homeostasis via salt secretion and osmoregulatory substance accumulation and preventing oxidative damage via increasing the activity of ROS scavenging system. These biological processes may be the most important responses of *Z. macrostachya* under salt stress. Collectively, our findings provided valuable insights into the molecular mechanisms and genetic underpinnings of salt tolerance in *Z. macrostachya*.

## Figures and Tables

**Table 1:** Element (Na, Ca, K, Cl) content (weight %) of the salt gland (basal cell and cap cell) in the control group and 300 mmol/L NaCl-treated group (24 h). Element content measured by X-ray energy spectrum. Results were presented as a percentage. Means (± SD) were calculated from three replications (*n* = 3) for each group. -: represents undetected.

Treatment (mmol/L)	Cell Type	Element Content (Weight %)			
		Na	Ca	K	Cl
0	Basal cell	-	-	29.89 ± 1.79	7.06 ± 1.03
	Cap cell	0.01 ± 0	6.07 ± 0.17	6.07 ± 0.48	13.33 ± 2.45
300	Basal cell	1.94 ± 0.21	1.10 ± 0.17	1.59 ± 0.32	0.58 ± 0.15
	Cap cell	0.30 ± 0.05	2.10 ± 0.37	0.95 ± 0.21	0.28 ± 0.08

## Supplementary Materials

The following are available online at https://www.mdpi.com/2223-7747/9/4/458/s1, Table S1: Unigenes annotated in NT database, Table S2: Information of the up- and down-regulated unigenes in *Zoysia macrostachya*, Table S3: The Gene Ontology (GO) enrichment results of the up-regulated genes in *Z. macrostachya*, Table S4: The Gene Ontology (GO) enrichment results of the down-regulated genes in *Z. macrostachya*, Table S5: Information of the qRT–PCR primers. Table S6. REVIGO analysis of up-regulated DEGs in *Z. macrostachya*.
